# Does the highly prevalent East Asian ALDH2 null variant magnify adverse effects of prenatal alcohol exposure on child development? A commentary

**DOI:** 10.1111/acer.70070

**Published:** 2025-05-09

**Authors:** Chloe Slaney, George Davey Smith

**Affiliations:** ^1^ MRC Integrative Epidemiology Unit University of Bristol Bristol UK

That maternal alcohol consumption during pregnancy may lead to adverse consequences for the offspring is well known, but it is less widely recognized that this problem may be magnified substantially in East Asian countries due to the high prevalence of *ALDH2* null variation. The paper by Miyake et al. (2025) in this journal leveraged data from a large Japanese population‐based cohort to investigate the joint associations of maternal alcohol use during pregnancy and maternal genotype on offspring development. We provide context for this study, highlight what we think is its critical finding and key questions that still need to be addressed before considering the potentially serious public health implications of this work.

Maternal alcohol use during pregnancy is associated with many adverse offspring outcomes (e.g., impaired neurodevelopment, facial dysmorphology, birth defects, growth deficiency) (Popova et al., 2023). These are broadly captured under the umbrella term fetal alcohol spectrum disorders (FASD), with fetal alcohol syndrome (FAS) at the severe end. However, like many conditions, FAS and FASD likely reflect continua, with their presentation and severity likely influenced by variable alcohol use, for example, timing and dose. FASD is associated with increased mortality (mean life expectancy for FAS individuals is 34 years old) and has severe social and economic consequences (Popova et al., 2023). Despite being preventable, FASD affects >1% of the population in 76 countries (Popova et al., 2023), with estimated prevalence rates mirroring rates of maternal alcohol use during pregnancy (Popova et al., 2017). For example, in East and Southeast Asia, maternal drinking during pregnancy (South Korea [21.4%], Cambodia [15.4%], Vietnam [12.0%], Japan [8.0%] and China [6.5%]) mirrors prevalence of FAS (per 10,000 births: 31.8, 22.8, 17.7, 11.8 and 9.6, respectively) (Popova et al., 2017). Importantly, prevalence rates of both FASD and alcohol use during pregnancy are likely under‐estimated. Factors which may contribute to under‐estimation include challenges in diagnosing FASD, under‐reporting of alcohol use (e.g., due to recall error and stigma), and unintentional drinking during pregnancy (due to unawareness of pregnancy early on). The latter is important considering that, across these East and Southeast Asian countries, estimates of women drinking have increased: current drinkers (1%–20% more in 2017 than 1990; using an inclusive definition of “any alcohol use in the past 12 months”) and heavy drinkers (3%–8% more in 2017 than 1990; excluding Japan, in which this decreased) (Manthey et al., 2019). Crucially, although FASD is caused by alcohol use during pregnancy, only a proportion of children who experience prenatal alcohol exposure (PAE) develop FASD. Identifying factors in addition to dose and duration of maternal alcohol consumption that influence FASD outcomes is therefore a public health priority.

One key factor suggested to impact FASD vulnerability is genetics. Evidence supporting this comes from both animal and human studies (Langevin et al., 2011; Sambo & Goldman, 2023). In humans, strong evidence comes from twin studies. In offspring that had PAE, monozygotic twins have 100% concordance for FAS and fetal alcohol effects, whereas dizygotic twins have 56%–64% concordance (Hemingway et al., 2018; Streissguth & Dehaene, 1993). While the limited sample size of these studies must be acknowledged (9 MZ and 39 DZ; 5 MZ and 11 DZ), they strongly support the idea that genetics—and specifically *fetal genetics*—play an important role in FASD. The precise genes involved in increasing (or reducing) the risk of FASD are unclear, but accumulating evidence implicates genes responsible for metabolizing ethanol and its toxic metabolite, acetaldehyde (Figure 1). Two well‐studied genes are Alcohol Dehydrogenase 1B (*ADH1B*), which encodes an enzyme that metabolizes ethanol into acetaldehyde, and Aldehyde Dehydrogenase 2 (*ALDH2*), which encodes an enzyme that metabolizes acetaldehyde and other aldehydes that are present in some foodstuffs or are by‐products of metabolic pathways (Chen et al., 2014). While negative effects of maternal alcohol consumption on the developing fetus were originally attributed to ethanol exposure, more recent work implicates acetaldehyde toxicity (Chen et al., 2014). As highlighted by Miyake et al., most human studies have focused on *ADH1B*, with less work done on *ALDH2*. This is despite the ALDH2*2 null variant being highly prevalent in East Asia (up to ~40%) and associated with severely reduced enzymatic activity (compared with ALDH2*1 homozygotes, ALDH2*2 heterozygotes have been reported to have up to ~80% reduced activity, and ALDH2*2 homozygotes have almost no activity), which causes an increased buildup of acetaldehyde following alcohol consumption (Miyake et al., 2025).

**FIGURE 1:**
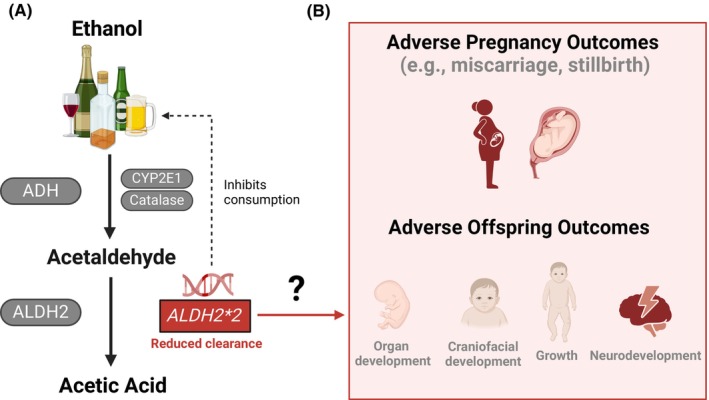
Postulated relationship between alcohol consumption and ALDH2*2, and potential adverse effects on pregnancy and offspring outcomes. (A) Ethanol is metabolized to acetaldehyde via ADH, and acetaldehyde is then metabolized to acetic acid via ALDH2 (its primary metabolizing enzyme). Additional enzymes are involved in metabolizing ethanol (CYP2E1 and Catalase), but for conciseness, these are not discussed further. Many East and Southeast Asian populations carry the ALDH2*2 genetic polymorphism (up to ~40%), which results in reduced clearance of acetaldehyde and consequently inhibits alcohol consumption due to its adverse effects (e.g., facial flushing and headaches). After an oral dose of ethanol (0.4 g/kg), peak blood acetaldehyde levels are substantially higher in ALDH2*2 homozygotes (79 μmol/L, *n* = 6) and heterozygotes (23 μmol/L, *n* = 29) than ALDH2*1 homozygotes (4 μmol/L, *n* = 33). (B) As acetaldehyde is toxic, the proposed theory suggests that following maternal alcohol consumption during pregnancy, a reduced ability to clear acetaldehyde (via mother and/or fetus ALDH2) may exacerbate the teratogenic effects following alcohol consumption. This theory needs to be tested using data from large population‐based cohorts. ADH, Alcohol Dehydrogenase; ALDH2, Aldehyde Dehydrogenase 2. Created with BioRender.com/f26a073.

After an oral dose of ethanol (0.4 g/kg), peak blood acetaldehyde levels are substantially higher in ALDH2*2 homozygotes (79 μmol/L, *n* = 6) and heterozygotes (23 μmol/L; *n* = 29) than in ALDH2*1 homozygotes (4 μmol/L, *n* = 33) (Mizoi et al., 1994). Mechanistically, it is unclear how *ALDH2* may impact fetal development. Nevertheless, animal studies suggest that ethanol and acetaldehyde can cross the placenta (Guerri & Sanchis, 1985). Although ethanol passes freely, acetaldehyde has a concentration gradient between the mother and fetus that varies with gestational age (Guerri & Sanchis, 1985). Increased fetal exposure to acetaldehyde in drinking women (via placental transfer and/or via fetal generation during ethanol metabolism), and its reduced clearance (due to *ALDH2*), may cause many aberrant effects on the fetal central nervous system (González‐Flores et al., 2024).

## THE CRUCIAL CONTRIBUTION OF MIYAKE ET AL.

Given the considerably raised levels of acetaldehyde following alcohol consumption in people with the *ALDH2* null variant in East Asia, we focus on the *ALDH2* genotype findings of Miyake et al. (2025). Leveraging data from a large Japanese population‐based cohort, they investigated the joint associations of maternal alcohol use during pregnancy and maternal genotype (*ADH1B* and *ALDH2*) on child development at age three in 1727 mother–child pairs. Specifically, participants were stratified into six groups based on alcohol use in pregnancy (three groups: never drank, quit drinking in early pregnancy, and current drinkers at second/third trimester of pregnancy) and either maternal *ADH1B* genotype (2 types: *2/*2 vs. *1/*1 + *1/*2) or maternal *ALDH2* genotype (2 types: *1/*1 vs. *1/*2). For *ADH1B* genotype, no substantial influences on the offspring were found. However, for *ALDH2* genotype, compared with mothers least at risk (ALDH2*1/*1 who did not drink during pregnancy), mothers with the ALDH2*2 polymorphism who were current drinkers had offspring with substantially higher risk of developmental impairments across all five domains assessed (communication, gross motor, fine motor, problem‐solving, and personal and social skills). As expected, no mothers who were homozygous for ALDH2*2 drank alcohol, and only 5 heterozygote mothers drank alcohol during pregnancy. What is most surprising and warrants further attention are the extremely large effects reported in this study (odds ratios ~10+), which were observed across all developmental domains. Given the small sample size, the confidence intervals are wide, and so there is a need for these findings to be replicated in larger population‐based birth cohorts in East Asia. Nevertheless, if these substantial effects replicate, they would have substantial public health implications.

Associations between maternal alcohol use during pregnancy and impaired offspring development could be *causal* or could reflect potential confounding, given that alcohol use is highly correlated with other lifestyle variables (e.g., smoking, diet, and other substance use). While smoking is unlikely to confound associations reported in Miyake et al. (as only 1 in 34 current drinkers also smoked during pregnancy), other confounders may be present. Mendelian randomization (MR) is a genetic epidemiological method that overcomes this challenge by using genetic variant(s) robustly associated with the exposure (alcohol use) as a proxy for the exposure (Davey Smith & Ebrahim, 2003). This approach is less susceptible to confounding as genetic variant(s) are randomly inherited from parents to offspring, making them less likely to be associated with confounders, and are fixed at conception, and therefore cannot be influenced by reverse causation (Davey Smith & Ebrahim, 2003). Previous studies have demonstrated the potential of using parent‐offspring MR to test the *causal* role of maternal pregnancy exposures on offspring outcomes (Davey Smith & Ebrahim, 2003). For example, MR studies support a causal role of maternal folic acid in preventing neural tube defects, consistent with randomized controlled trials of maternal folate supplementation (Davey Smith & Ebrahim, 2003). Importantly, using offspring and paternal genotype confirmed that it is *maternal* folic acid levels that play a key role in neural tube defects (Davey Smith & Ebrahim, 2003). These studies also highlight the importance of using paternal genotype as a negative control when testing intrauterine exposures on offspring outcomes, with the expectation that there would be no effect of paternal genotype. While there have been MR studies of maternal ethanol exposure (using *ADH* genotypes) on offspring outcomes (Mamluk et al., 2021), to our knowledge, there are no MR studies of acetaldehyde (using *ALDH2* genotype) on offspring outcomes. The paper by Miyake et al. is the closest attempt at testing this in humans. However, in contrast to the folate example above where maternal genotype is relevant, fetal *ALDH2* genotype could be having a more important influence in associations between maternal alcohol consumption and offspring development. Studies that include fetal and maternal genotype can directly test this hypothesis.

## KEY QUESTIONS THAT STILL NEED TO BE ADDRESSED

### PAE and offspring development: Is maternal or fetal *ALDH2* genotype key?

Although Miyake et al. report the combined associations of maternal ALDH2*2 and alcohol consumption on offspring development in children, they did not test the effect of fetal genotype. Therefore, the relative importance of maternal versus fetal genotype remains to be addressed. While twin studies suggest fetal genetics play a key role in FASD symptoms (Hemingway et al., 2018; Streissguth & Dehaene, 1993), animal studies suggest both maternal and offspring *ALDH2* genotype likely play an important role in offspring development (Langevin et al., 2011). In mice, this has been shown in the context of Fanconi anemia, a condition with defective DNA repair. Langevin et al. (2011) showed that ALDH2*2 homozygote pregnant dams could not support knock‐out offspring (*ALDH2* ^−/−^ and *FANCD2* ^−/−^) resulting in embryonic death. Knock‐out embryos of *ALDH2* heterozygote pregnant dams (who preserve some *ALDH2* activity) were hypersensitive to developmental deformities following maternal alcohol exposure (Langevin et al., 2011), suggesting fetal *ALDH2* is also important. Miyake et al. state that the influence of genetic polymorphisms in children could not be evaluated, despite child genotype data being generated in their cohort. We assume that this is because only five mothers with ALDH2*2 were current drinkers, or the child genotype data were not yet available when Miyake et al. conducted their analysis. We believe that even with the small sample size, it would be useful to stratify fetal genotype by maternal genotype to get a sense of the relative importance of fetal acetaldehyde metabolism. This is critical as it may reveal even larger developmental risk in ALDH2*2 homozygote offspring. More broadly, it would be useful to examine fetal genotype of all current drinkers (*n* = 34) and heterozygote mothers who quit in early pregnancy (*n* = 184), as there will be ALDH2*2 carrying offspring in these groups. Additionally, as highlighted in the folic acid example above, paternal genotype can be used as a negative control, and if available, should be incorporated. Going forward, larger studies which have information on maternal, fetal, and paternal genotype are required.

Notably, there is some evidence that alcohol and aldehyde metabolizing enzymes change in abundance and/or gene expression levels across fetal development and tissues (e.g., liver versus brain) (Alnouti & Klaassen, 2008). Speculatively, assuming fetal enzymatic activity increases throughout gestation, one may expect fetal genotype to play a larger role in preventing aberrant effects at later stages in gestation, compared with earlier stages where maternal genotype may be more relevant. As Miyake et al. found joint associations of maternal *ALDH2* genotype and drinking in *mid‐pregnancy* on offspring development, it could be that fetal genotype has a more important influence at this developmental stage. Future studies should investigate the role of fetal and maternal *ALDH2* genotype on associations between PAE and offspring development, taking into consideration the timing and amount of alcohol exposure (e.g., first vs. third trimester) *and* specific outcomes (e.g., cognition and growth).

### Alcohol use, acetaldehyde, and offspring development: Is there a causal link?

Determining whether maternal alcohol use *causes* adverse offspring outcomes is challenging due to potential confounding (e.g., maternal smoking and diet). MR can overcome this challenge by using genetic variants robustly associated with alcohol use as a proxy. Maternal ALDH2*2*2 provides an ideal proxy for “no drinking” (as illustrated in Miyake et al. in whose data no ALDH2*2*2 women drink, likely due to its adverse effects). If alcohol is *causal*, we would expect reduced risk of adverse offspring outcomes in ALDH2*2*2 mothers, compared with ALDH2*1*1. For MR analysis, only *maternal ALDH2* genotype—which is used as a proxy for maternal drinking—is relevant. Subsequently, it will be important to test interactions of maternal and fetal *ALDH2* genotype and alcohol intake (nondrinkers, intermediate, and heavy drinkers) on offspring outcomes, which may provide insight into the role of acetaldehyde exposure. This approach has been successfully applied in cancer (Lewis & Davey Smith, 2005) and would be useful in the present context.

### PAE and adverse pregnancy outcomes: Does *ALDH2* genotype play a role?

Additionally, another important question that needs to be addressed is the effect of ALDH2*2 on associations between PAE and adverse *pregnancy* outcomes (e.g., miscarriage, stillbirth, preterm birth, sudden infant death, and low birthweight). PAE is a risk factor for these adverse outcomes (Popova et al., 2017), which may reflect fetal toxicity. As these outcomes are of clear importance for public health, they should also be looked at in relation to maternal and fetal *ALDH2* genotype.

### Maternal alcohol use during breastfeeding and offspring development: What is the impact of ALDH2*2?

As alcohol freely passes into breast milk (and acetaldehyde may enter milk) (Guerri & Sanchis, 1986), it is possible that maternal drinking during breastfeeding may impact child growth and development (May et al., 2016), with effects modulated by ALDH2*2. Compared with PAE, there is much less evidence on the effects of alcohol exposure from breast milk on offspring development. Nevertheless, using data from four South African active‐case ascertainment FASD studies (1047 mother‐offspring pairs), May et al. (2016) reported that after adjusting for potential confounders (including prenatal drinking), children whose mothers drank postpartum and breastfed were 6.4 times more likely to have FASD than breastfed children whose mothers abstained from alcohol (May et al., 2016). Therefore, there is a need for studies to test this association, incorporating both maternal and child *ALDH2* genotype, in large East and Southeast Asian cohorts.

## POTENTIAL IMPLICATIONS

Miyake et al.'s findings may have critical implications, given that ALDH2*2 is highly prevalent in several East and Southeast Asian countries, and that many women of reproductive age are consuming alcohol. First, ALDH2*2 is present in ~560 million people (~8% globally) (Chen et al., 2014). Stratified by region, ALDH2*2 is estimated to be highly prevalent in Japan (up to ~30%), but also in China (up to ~40%), South Korea (up to ~25%), Vietnam (~10%–18%) and Cambodia (~15%) (Li et al., 2009) (although given the small sample sizes for Vietnam and Cambodia, more ALDH2*2 prevalence studies are needed in these populations). Second, many women consume alcohol during pregnancy. Based on prevalence estimates from Popova et al. (2017), alcohol consumption during pregnancy is less common in Japan (8.0%) and China (6.5%), but it is higher in South Korea (21.4%), Cambodia (15.4%), and Vietnam (12.0%) (Popova et al., 2017). Third, while many women stop drinking alcohol during pregnancy, globally 48% of pregnancies are unplanned, with similar estimates observed when restricting to East and Southeast Asia (53%) (Bearak et al., 2020). Moreover, as mentioned earlier, increased trends of women drinking in these countries (Manthey et al., 2019) may result in many fetuses being unintentionally exposed to alcohol during early development. Taken together, there are many East and Southeast Asian populations for whom this work could have crucial public health implications.

## CONCLUSION

To summarize, Miyake et al. present data relevant to a pressing global public health issue that has not yet been adequately addressed in human studies. They report substantial effects of maternal ALDH2*2 on associations between alcohol use in pregnancy and offspring neurodevelopment. As many people have this variant in East and Southeast Asia, and alcohol drinking in women is increasing in these countries, these findings could have serious implications. Going forward, there is a need to replicate and expand these findings in large population‐based cohorts and to disentangle the relative importance of maternal versus fetal *ALDH2* genotype. Data on paternal genotype, which can be used as a negative control, will also be highly valuable.

## FUNDING INFORMATION

CS and GDS work within the MRC Integrative Epidemiology Unit at the University of Bristol, which is supported by the Medical Research Council (MC_UU_00032/6 and MC_UU_00032/1).

## CONFLICT OF INTEREST STATEMENT

The authors have no conflict of interest to declare.
